# A novel *CBL-B^flox/flox^* mouse model allows tissue-selective fully conditional *CBL/CBL-B* double-knockout: CD4-Cre mediated *CBL/CBL-B* deletion occurs in both T-cells and hematopoietic stem cells

**DOI:** 10.18632/oncotarget.9812

**Published:** 2016-06-03

**Authors:** Benjamin Goetz, Wei An, Bhopal Mohapatra, Neha Zutshi, Fany Iseka, Matthew D. Storck, Jane Meza, Yuri Sheinin, Vimla Band, Hamid Band

**Affiliations:** ^1^ Eppley Institute for Research in Cancer and Allied Diseases, University of Nebraska Medical Center, Omaha, NE 68198, USA; ^2^ Departments of Biochemistry and Molecular Biology, University of Nebraska Medical Center, Omaha, NE 68198, USA; ^3^ Department of Pathology and Microbiology, University of Nebraska Medical Center, Omaha, NE 68198, USA; ^4^ Department of Genetics, Cell Biology and Anatomy, University of Nebraska Medical Center, Omaha, NE 68198, USA; ^5^ Departments of Pharmacology and Experimental Neuroscience, College of Medicine, University of Nebraska Medical Center, Omaha, NE 68198, USA; ^6^ Department of Biostatistics, College of Public Health, University of Nebraska Medical Center, Omaha, NE 68198, USA; ^7^ Fred and Pamela Buffet Cancer Center, University of Nebraska Medical Center, Omaha, NE 68198, USA

**Keywords:** CBL-family ubiquitin ligases, T-cell, CD4, hematopoietic stem cell, conditional knockout

## Abstract

CBL-family ubiquitin ligases are critical negative regulators of tyrosine kinase signaling, with a clear redundancy between CBL and CBL-B evident in the immune cell and hematopoietic stem cell studies. Since CBL and CBL-B are negative regulators of immune cell activation, elimination of their function to boost immune cell activities could be beneficial in tumor immunotherapy. However, mutations of CBL are associated with human leukemias, pointing to tumor suppressor roles of CBL proteins; hence, it is critical to assess the tumor-intrinsic roles of CBL and CBL-B in cancers. This has not been possible since the only available whole-body CBL-B knockout mice exhibit constitutive tumor rejection. We engineered a new CBL-B^flox/flox^ mouse, combined this with an existing CBL^flox/flox^ mouse to generate CBL^flox/flox^; CBL-B^flox/flox^ mice, and tested the tissue-specific concurrent deletion of CBL and CBL-B using the widely-used CD4-Cre transgenic allele to produce a T-cell-specific double knockout. Altered T-cell development, constitutive peripheral T-cell activation, and a lethal multi-organ immune infiltration phenotype largely resembling the previous Lck-Cre driven floxed-CBL deletion on a CBL-B knockout background establish the usefulness of the new model for tissue-specific CBL/CBL-B deletion. Unexpectedly, CD4-Cre-induced deletion in a small fraction of hematopoietic stem cells led to expansion of certain non-T-cell lineages, suggesting caution in the use of CD4-Cre for T-cell-restricted gene deletion. The establishment of a new model of concurrent tissue-selective CBL/CBL-B deletion should allow a clear assessment of the tumor-intrinsic roles of CBL/CBL-B in non-myeloid malignancies and help test the potential for CBL/CBL-B inactivation in immunotherapy of tumors.

## INTRODUCTION

Tight regulation of T-cell activation and immunological tolerance are essential to allow the body to mount effective defense against foreign antigens and to provide immune surveillance against cancer without evoking autoimmunity. A key mechanism to prevent autoimmune consequences of peripheral T-cell activation during immune responses is the imposition of a requirement for concurrent signals emanating from the T-cell receptor (TCR) recognition of an antigen and those generated by the engagement of co-stimulatory molecules [1]. Engagement of the TCR in the absence of co-stimulatory molecules results in cell-intrinsic functional inactivation known as anergy [2]. During physiological immune responses, the function of co-stimulatory receptors and ligands is counter-balanced by negative co-stimulatory molecules on T-cells, such as CTLA4, and inhibitory ligands on APCs/target cells [1]. Recent work has established that active editing of immune mechanisms in tumors tilts this balance towards inhibitory rather than co-stimulatory signaling in T-cells, providing a major mechanism of immune escape used by tumors [3]. Indeed, antibody-based blockade of inhibitory pathways (e.g., anti-CTLA4) is emerging as a viable form of immunotherapy [4].

The CBL-family ubiquitin ligases (E3s) are essential negative regulators of T-cell activation and mediate induction of immune anergy/tolerance programs [5]. The CBL-family proteins function as E3s directed specifically at protein tyrosine-kinase (PTK) signaling pathways activated by stimulation through a number of cell surface receptors, including the TCR [6]. This function involves a highly conserved mechanism in which the N-terminal tyrosine kinase-binding (TKB) domain of CBL proteins binds to specific phosphotyrosine-containing motifs on receptor or non-receptor PTKs or adaptor proteins phosphorylated upon receptor-induced PTK activation [7]. Once recruited, CBL proteins are phosphorylated, which facilitate the binding of a ubiquitin conjugating enzyme (E2) and juxtaposes the E2 enzyme closer to the TKB domain-bound PTKs [8]. CBL and CBL-B, but not the epithelial-restricted CBL-C, also contain homologous C-terminal extensions that include an extensive proline-rich region and specific tyrosine phosphorylation sites for association with signaling proteins [9–11]. These mechanisms contribute to the negative regulation of PTK-coupled surface receptor signals that involve ubiquitination-dependent lysosomal targeting and/or proteasomal degradation of receptors and their associated signaling proteins [12–14].

Genetic studies using a whole-body CBL-B knockout mouse model demonstrate that CBL-B plays an essential role in coupling T-cell activation to the requirement for CD28-mediated co-stimulation [9, 15, 16]. In addition, CBL-B promotes destabilization of the immunological synapse by negatively regulating integrin activation [11]. CBL-B is also a critical regulator of the anergy induction program and becomes transcriptionally up-regulated under T-cell anergy-inducing conditions [17, 18]. As a result, CBL-B null mice exhibit increased susceptibility to spontaneous or induced autoimmunity [19]. Although CBL-B is expressed in immature thymocytes, CBL-B null mice show no detectable alterations in thymic development [15, 19].

CBL-B-null mice exhibit enhanced anti-tumor immunity in spontaneous and transplanted tumor models [5, 20]. However, the elucidation of mechanisms of enhanced anti-tumor ability in the currently-available CBL-B null mouse models has been challenging since all immune and non-immune cells lack CBL-B expression. Adoptively-transferred CBL-B null CD8 T-cells or NK cells have been shown to exhibit an anti-tumor effect in mouse studies [21–24]. Based on these studies, downregulation of CBL-B in human T-cells has been shown to enhance their tumor killing abilities [25]. Recent studies suggest an increased expression of CBL-B within tumor-associated immune component, consistent with a role in mediating immune tolerance to tumors [26]. While these studies raise the prospect of inactivating CBL-B for immunotherapy of tumors, this has not received much consideration.

Whole-body CBL-null mice exhibit altered thymocyte development with increased thymocyte numbers and enhanced positive selection of mature CD4^+^ T-cells [27]. While CBL^−/−^ thymocytes exhibited hyper-activated ZAP70 and MAPK, curiously they also showed reduced activity of PI3K and PLCγ1, and impaired activation-induced TCR down-modulation [27, 28]. Even though CBL can interact with many of the same substrates as CBL-B, such as PI3K, Vav1 and CrkL, CBL-deficient mice display relatively normal peripheral T-cell function to the extent studied [29]. Thus, the *in vivo* function of CBL in peripheral T-cells remains incompletely characterized.

While CBL-B^−/−^ mice display increased sensitivity to development of autoimmunity and CBL^−/−^ mice show normal peripheral T-cell function, CBL/CBL-B double knockout is embryonic lethal and induction of Cre-mediated deletion of a floxed CBL allele by Lck-Cre (deletion at the double negative (DN) stage of thymocyte development) on a CBL-B null background led to severe spontaneous autoimmune organ infiltration, splenomegaly, and auto-antibodies leading to death between 12 and 16 weeks of age [30]. CBL and CBL-B double-deficient T-cells exhibit even higher proliferation compared to CBL-B^−/−^ T-cells when stimulated with an anti-CD3 antibody. Combined deletion of CBL and CBL-B also leads to a more severely altered thymic development [31]. Aside from studies of T-cells, redundant functional roles of CBL and CBL-B have also emerged from a number of *in vitro* studies and genetic studies in other systems [32–35]. Deletion of floxed CBL with murine mammary tumor virus (MMTV)-Cre on a CBL-B null background led to a myeloproliferative disease due to CBL deletion in hematopoietic stem cells (HSCs), but such a phenotype was not observed when CBL alone or CBL-B alone were deleted [36, 37]. Using the same model, we have recently observed a redundant requirement of CBL and CBl-B in mammary gland development (Mohapatra B, Zutshi N, An W, Goetz B, Arya P, Bielecki T, Storck M, Meza J, Band V, Band H. An essential role of CBL and CBL-B ubiquitin ligases in mammary stem cell maintenance. submitted.).

In contrast to a potentially pro-oncogenic role of CBL proteins by promoting immune tolerance associated with tumorigenesis, a potentially opposite role of CBL proteins as tumor suppressors has emerged in the context of leukemogenesis. Mutations clustered in the linker region or RING finger domain of CBL, and rarely CBL-B, which abrogate E3 activity, have been identified in a subset of patients with myelodysplastic syndrome/myeloproliferative neoplasms (MDS/MPN), chronic myelomonocytic leukemia or juvenile myelomonocytic leukemia [38–44]. Loss of CBL expression was shown to accelerate BCR-abl induced myeloid leukemogenesis in a mouse model [45]. Mice with an inactivating RING finger domain mutation in CBL also exhibit a leukemic disease when the WT CBL gene was deleted [46]. A more rapid leukemic disease was observed upon conditional CBL deletion, using MMTV-Cre, on a CBL-B null background thus supporting a redundant but essential role of CBL and CBL-B as tumor suppressors in the context of myeloid leukemogenesis [36, 37]. Whether or not CBL proteins have a role during tumorigenesis of non-myeloid lineages remains unknown; however, recent studies suggest a potentially pro-oncogenic role of CBL as its expression was found to be higher in breast cancer and depletion of CBL/CBL-B reduced tumorigenicity or metastasis of breast cancer cells in a nude mouse model [47, 48]. These suggestive findings make it vital to design models where tissue-specific and tumor-intrinsic deletion of CBL and/or CBL-B can be induced to assess non-myeloid cell and tumor cell-intrinsic roles of CBL proteins.

It is currently not feasible to test the functional redundancy of CBL proteins in specific populations of T-cells using existing models because their generalized CBL-B deficiency leads to altered and/or enhanced function of all T-cell subsets and other immune cells, including B cells [49], macrophages [50, 51], mast cells [52], neutrophils [53, 54], and NK cells [55]. Here, we describe the first CBL-B^flox/flox^ mouse which allows conditional CBL and CBL-B deletion in a cell type-specific manner. By crossing this new mouse strain with the previously generated CBL^flox/flox^ mouse and to a CD4-Cre transgen (Tg(Cd4-cre)1Cwi/BfluJ), we obtained concurrent CBL and CBL-B double knockout (DKO) in all T-cell subsets, resulting in altered T-cell development, widespread organ infiltration by immune cells and rapid lethality, consistent with a redundant functional role of CBL and CBL-B. Surprisingly, however, deletion of CBL and CBL-B was found to also occur in HSCs, resulting in their expansion and altered hematopoiesis, a result that brings into question the widespread use of CD4-Cre as driver of T-cell-specific gene deletion and suggests a cautious interpretation of studies with this Cre driver, especially in contexts where deletion results in loss of expression of a negative regulatory gene product.

## RESULTS

### Generation of CBL-B floxed mice

**Figure 1:**
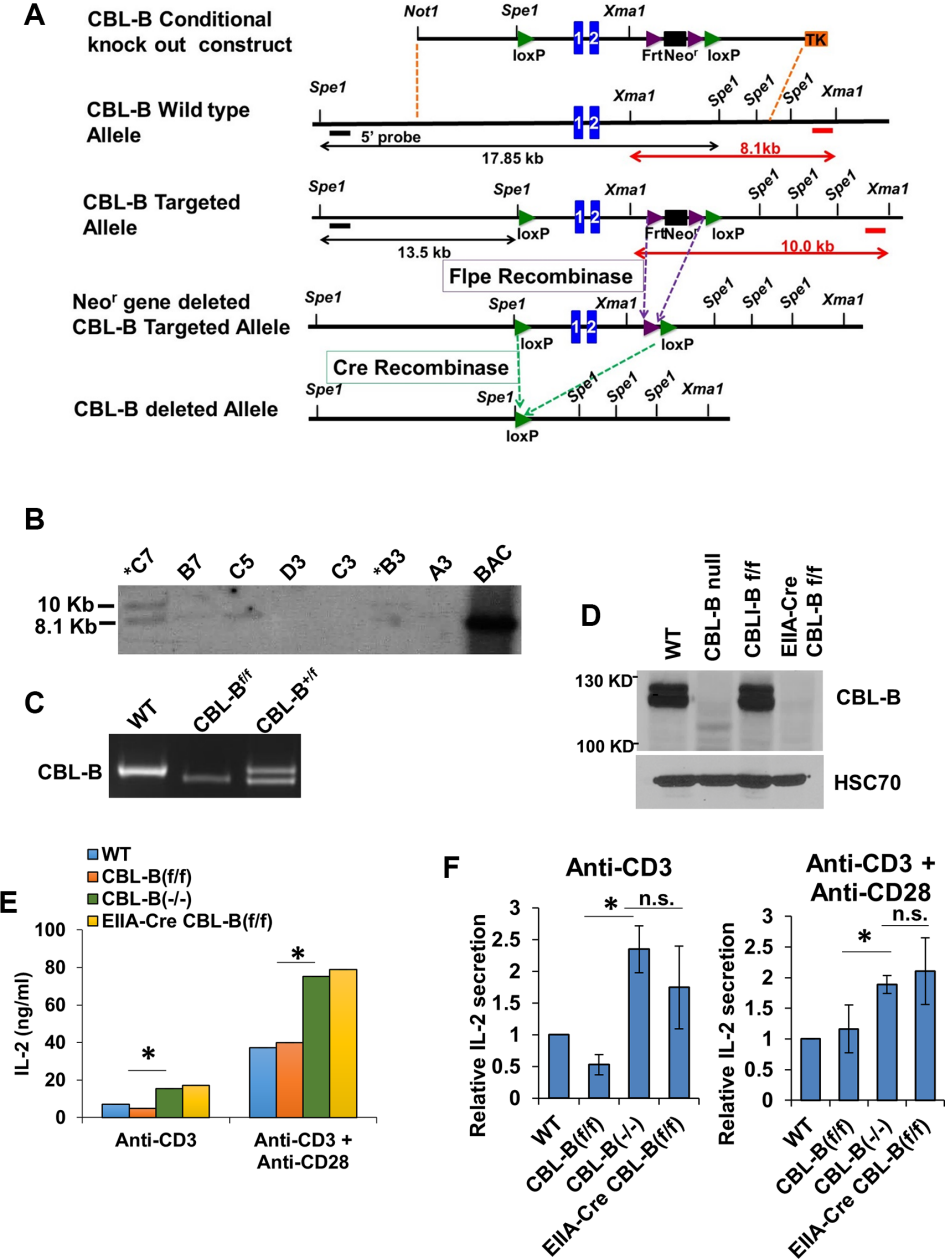
Generation of the CBL-B floxed allele (**A**) Strategy for generating the CBL-B floxed targeting vector and CBL-B floxed (targeted) allele. Blue boxes represent exons. The 5′ external probe for Southern Blotting is indicated by the thick black line and 3′ external probe is displayed by thick red lines. The predicted length of Southern fragments is indicated with double arrow lines. (**B**) Targeted events were identified by Southern analysis of Xma1- digested genomic ES cell DNAs with a 3′ flanking probe. There is a 1.9 KB insertion of the loxP-Frt cassette after proper targeting. B6 ES clones identified after southern blot. (**C**) Confirmation of the genotype of CBL-B floxed animals generated in our lab using allele-specific PCR primers detecting the floxed CBL-B allele correspond to the insert region containing the loxP site. (**D**) Western Blot validating the deletion of CBL-B in EIIA-Cre CBL-B floxed mice. Splenocytes were collected from mice with indicated genotype and total protein lysate were blotted for CBL-B and HSC70. (**E**–**F**) IL-2 ELISA. Splenocytes were collected from mice with indicated genotype and plated for 48 hours before medium were collected for IL-2 quantification. (E) is one representative experiment and (F) is pooled data from three experiment and shown as relative level normalized to WT control. Data shown are mean +/− SD. ns, *p* ≥ 0.05; *p ≤ 0.05; **p ≤ 0.01; ***p ≤ 0.001; ****p ≤ 0.0001.

Since a regular knockout strategy targeting exons 1 and 2 of CBL-B gene is known to yield mice that completely lack CBL-B protein expression [15], we chose to target the same exons to generate CBL-B^flox/flox^ mice as described in the Materials and Methods section, using the CBL-B conditional knockout construct shown in Figure 1A. Successfully-targeted embryonic stem cell clones and chimeric mice were identified based on the generation of a 10 kb fragment in Southern Blots of *Xma1* digested genomic DNA (Figure 1B). Successfully-targeted founder mice were crossed with a Flp recombinase strain to help excise the Neomycin-resistance cassette. The heterozygous floxed mice were used to generate the CBL-B^flox/flox^ mice whose genotype was confirmed by PCR, with the floxed allele generating a 750 bp fragment while the WT allele generates a 850 bp fragment (Figure 1C). To confirm that the inserted floxed sites were susceptible to Cre cleavage *in vivo*, we crossed these mice with an EIIA-Cre transgenic line and assessed the deletion of floxed CBL-B allele in germline by Western blotting of splenocytes demonstrating a complete loss of CBL-B protein expression similar to that seen with positive control CBL-B (−/−) splenocytes, while CBL-B protein expression in CBL-B^flox/flox^ and WT mouse splenocytes was comparable (Figure 1D), excluding any negative impact of the introduced flox sites themselves on CBL-B expression.

To further verify the functional impact of CBL-B deletion in the new conditional CBL-B deletion model, splenocytes isolated from CBL-B^flox/flox^; EIIA-Cre mice were subjected to stimulation using an anti-CD3 antibody with or without an anti-CD28 antibody. It has been established that CBL-B-deficient T-cells secrete higher levels of IL-2 upon stimulation with an anti-CD3 antibody alone, or with an anti-CD3 plus anti-CD28 stimulation [15]. Indeed, anti-CD3 or anti-CD3 plus anti-CD28 stimulation induced higher levels of IL-2 production in CBL-B (−/−) T-cells from CBL-B^flox/flox^; EIIA-Cre mice similar to the increase in IL2 production seen using T-cells from conventional CBL-B (−/−) mice (Figure 1E and 1F). Collectively, these results establish that we have engineered a CBL-B^flox/flox^ model that does not affect CBL-B expression in the absence of Cre-mediated gene deletion and is fully amenable to Cre-mediated gene deletion *in vivo*, recapitulating the functional impact of whole-body CBL-B deletion on T-cells previously reported [15].

### CD4-Cre induced CBL/CBL-B deletion leads to strong hematological phenotype

**Figure 2:**
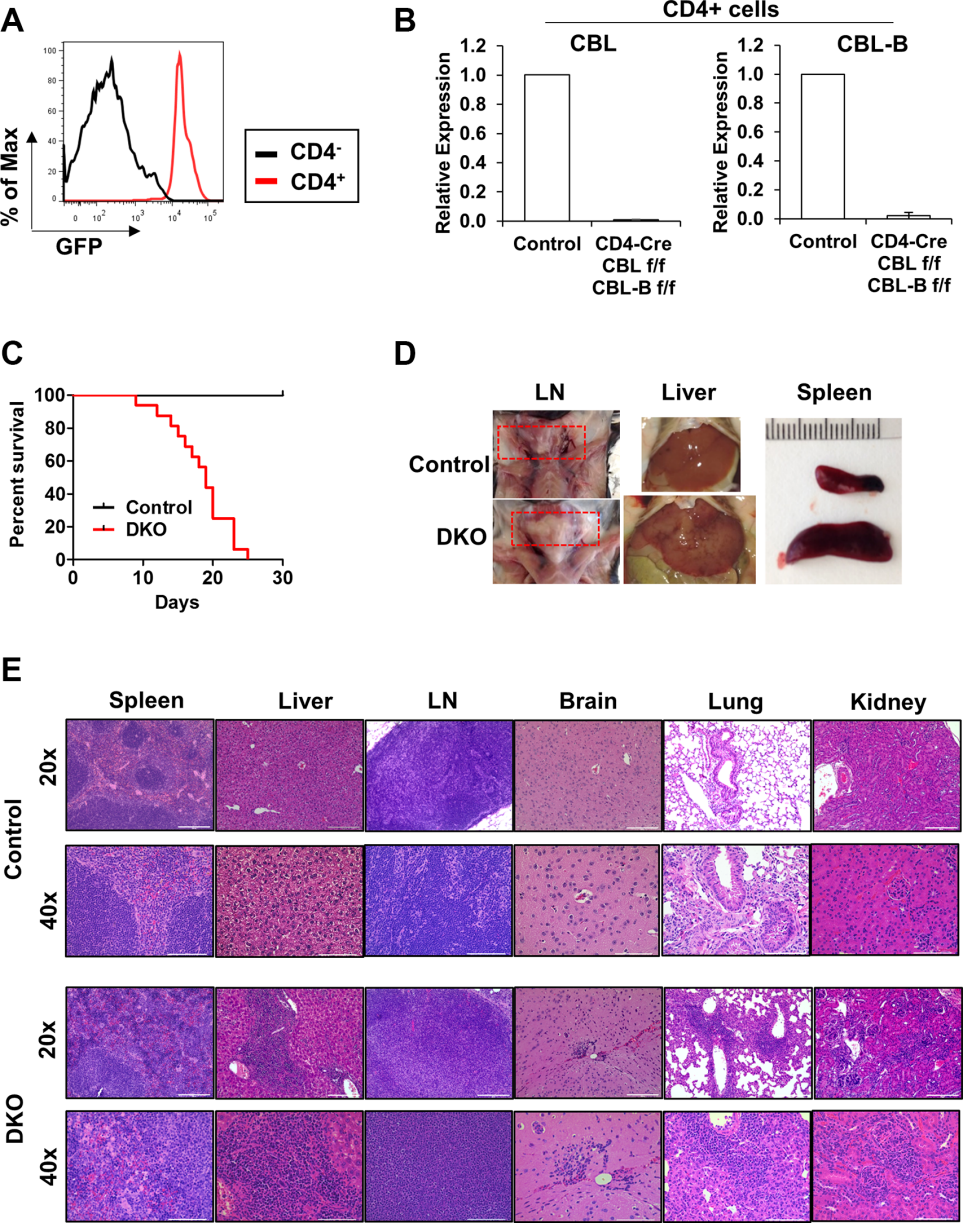
CD4-Cre induced CBL/CBL-B deletion leads to strong hematological phenotype (**A**) FACS analysis of splenocytes from DKO mice for the expression of GFP in CD4^+^ or CD4^−^ cells. (**B**) mRNA expression levels of CBL (left) and CBL-B (right) were analyzed in FACS-sorted CD4+ cells of Control or DKO mice by quantitative real-time PCR. (**C**) Kaplan-Meyer survival curve of Control and DKO mice; *n* = 16. (**D**) Representative photos demonstrating lymph node hyperproliferation and hepatosplenomegaly. Red rectangle indicates lymph nodes (**E**) Representative H & E stained liver, spleen, lymph node (LN), brain, lung and kidney sections. Bar for 20× and 40× images represents 200 μm and 100 μm respectively.

Previously, CBL/CBL-B double-KO in T-cells using Lck-Cre mediated deletion of CBL on a whole-body CBL-B KO was found to produce a spontaneous inflammatory disease that was eventually lethal (within 25 weeks of age), compared to a lack of inflammatory phenotype in the parental CBL-B-null mouse strain [15]. In our effort to examine the impact of T-cell specific, concurrent deletion of CBL and CBL-B, we generated CBL^flox/flox^; CBL-B^flox/flox^ mice using the previously generated CBL^flox/flox^ mice [30] and further crossed these to CD4-Cre transgenic mice to generate CBL^flox/flox^; CBL-B^flox/flox^; CD4-Cre mice for conditional deletion of CBL and CBL-B specifically in T-cells (referred to as DKO mice hereafter). We also introduced a dual-reporter of Cre-mediated gene deletion in which Rose-26 locus encoded membrane-localized td-Tomato (red) and GFP that are expressed prior to and after successful Cre-mediated deletion of floxed sequence cassettes respectively [56]. This reporter system provided a handy tool to identify cells that have undergone Cre-mediated deletion (GFP+). FACS analysis identified a substantial increase in GFP expression in CD4+ cells of the spleen, and real-time PCR analysis verified the deletion of CBL and CBL-B in these cells (Figure 2A and 2B).

The CBL^flox/flox^; CBL-B^flox/flox^; CD4-Cre+ (DKO) pups were born at expected Mendelian ratios (around 50% when crossed CBL^flox/flox^; CBL-B^flox/flox^ with CBL^flox/flox^; CBL-B^flox/flox^; CD4-Cre+). These mice however became moribund starting as early as 10 weeks of age and all became moribund and required euthanasia by 25 weeks (Figure 2C). Examination of 10-week old female DKO mice revealed the development of lymphomegaly and hepatosplenomegaly in all animals (*n* = 11) (Figure 2D). Hematoxylin & Eosin staining of formalin-fixed and paraffin-embedded sections showed a high degree of immune cell infiltration in multiple organs examined (Figure 2E). Histological analysis revealed intense immune cell aggregates and features of extramedullary hematopoiesis in the liver, perivascular immune clusters in the kidney, perivascular immune aggregates and signs of acute and chronic inflammation in the lung, as well as perivascular immune clusters in the brain. Pathological changes observed in lymphoid tissues includes enlargement of follicle size and less defined white pulp in the spleen, and increased medullary area in lymph nodes. The histopathology of the heart and intestine were normal (data not shown). These data show that CD4-Cre directed CBL/CBL-B DKO leads to a severe and lethal spontaneous autoimmune/inflammatory phenotype.

### CD4-Cre induced CBL/CBL-B DKO alters T-cell development and peripheral T-cell activation

**Figure 3:**
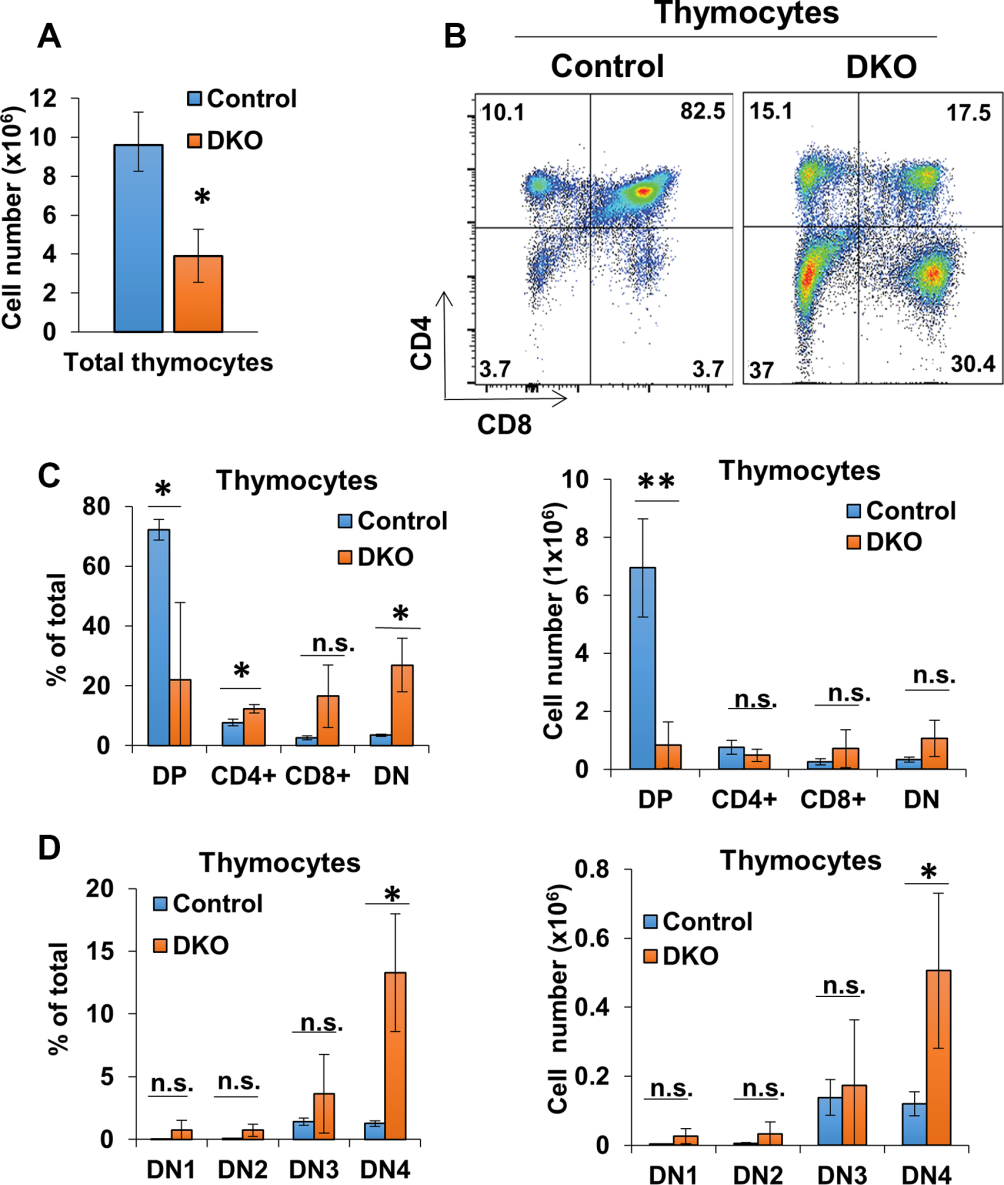
CD4-Cre induced CBL/CBL-B deletion leads to altered thymocyte development (**A**) Mean values of cell numbers from thymuses of 10-week old Control and DKO mice; *n* = 3. (**B**) Representative dot plot FACS analysis of anti-CD4 and anti-CD8 stained thymocytes. (**C**) FACS analysis of CD4CD8 DP, single-positive, and DN thymocyte populations for percentage of total thymocytes (left) and cell number (right); *n* = 3. (**D**) Flow analysis of DN thymocyte populations for percentage of total thymocytes (left) and cell number (right); *n* = 3. DN cells are gated (DN1: CD117^+^ CD44^+^ CD25^−^, DN2: CD117^+^ CD44^+^ CD25^+^, DN3: CD117^−^ CD44^−^ CD25^+^, DN4: CD117^−^ CD44^−^ CD25^−^). Data shown are mean +/− SD. ns, *p* ≥ 0.05; *p ≤ 0.05; **p ≤ 0.01; ***p ≤ 0.001; ****p ≤ 0.0001.

Since CBL family proteins are known to function as critical negative regulators of signaling in T-cells [14], we examined the effect of CD4-Cre induced DKO on T-cells within lymphoid tissues. 10-week old female DKO mice exhibited shrunken thymuses coincided with a marked reduction in the overall thymocyte numbers compared to control (CBL^flox/flox^; CBL-B^flox/flox^ mice without CD4-Cre) mice (Figure 3A). Examination of different thymocyte subpopulations in DKO mice revealed a diminished CD4 and CD8 double-positive (DP) thymocyte population; however, there was an increase in the percentage of CD4/CD8 double-negative (DN) thymocytes and a substantial, albeit not statistically-significant (with the exception of CD4), skewing of the relative percentages and absolute numbers of single-positive populations compared to control (Figure 3B and 3C). Compared to controls, the percentage of CD4+ thymocytes was modestly higher however this was not reflected in the overall CD4+ thymocyte numbers (Figure 3B and 3C). Further dissection of the DN population into DN1-4 subpopulations (based on the markers CD25, CD44, and CD117) [57] revealed an increase in DN4 population in DKO thymuses compared to control (Figure 3D). Altogether these data show that concurrent conditional deletion of CBL and CBL-B in T-cells using CD4-Cre leads to marked alterations in thymocyte development with a decrease in total double-positive thymocytes, an increase in double-negative thymocytes, and a skewing to CD4+ thymocyte proportions.

**Figure 4:**
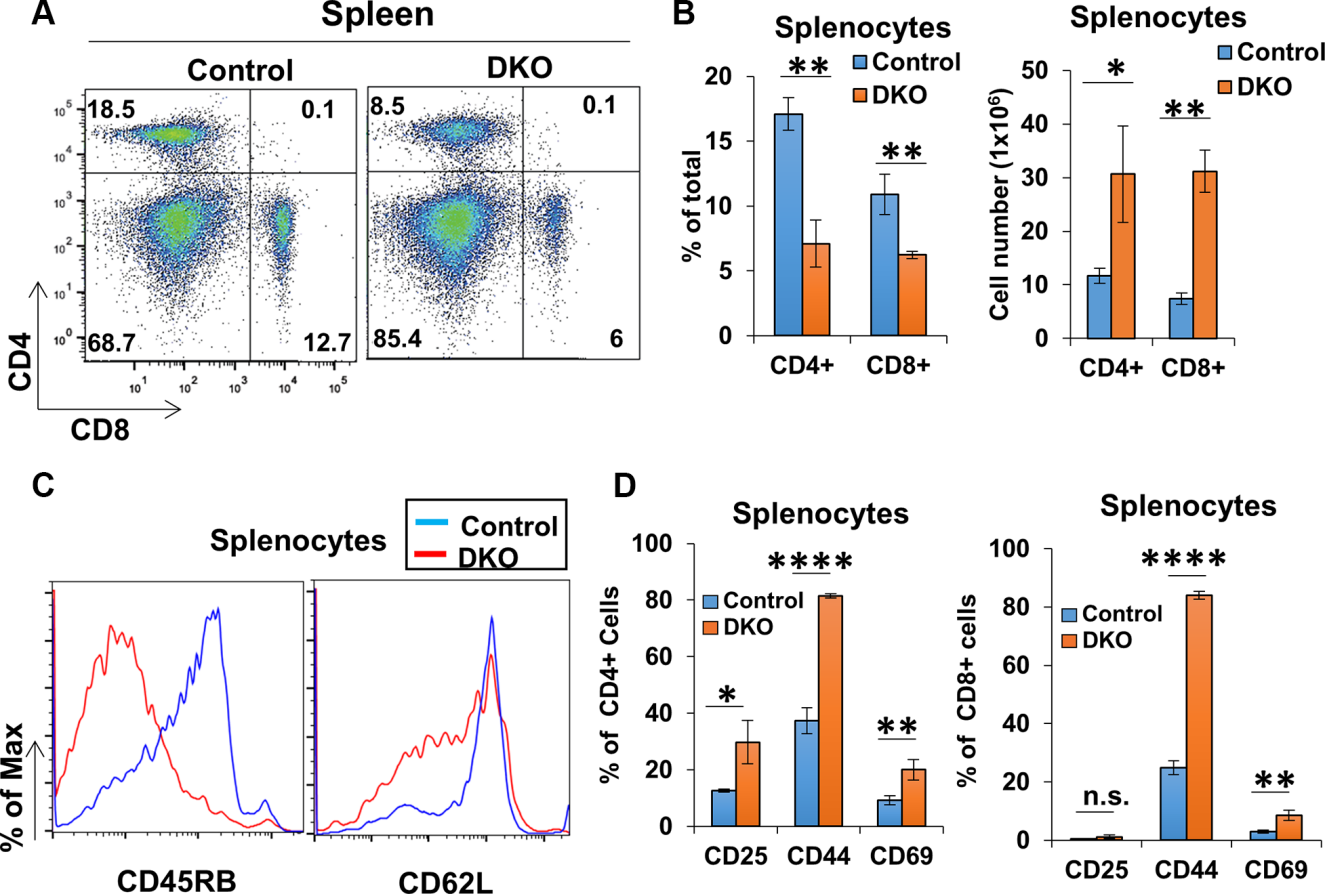
CD4-Cre induced CBL/CBL-B deletion leads to altered splenic T-cell activation status (**A**–**B**) Representative FACS dot plots (A) and mean values of percentage of total splenic populations stained with anti-CD4 and anti-CD8 (B) in Control and DKO mice; *n* = 3. (**C**) Representative histograms for FACS analysis of marker expression for CD4+ gated splenic population. (**D**) Quantification of the percentage of cells positive for markers CD25, CD44, and CD69 in splenic CD4+ (left) and CD8+ (right) gated populations; *n* = 3. Data shown are mean +/− SD. ns, *p* ≥ 0.05; *p ≤ 0.05; **p ≤ 0.01; ***p ≤ 0.001; ****p ≤ 0.0001.

**Figure 5:**
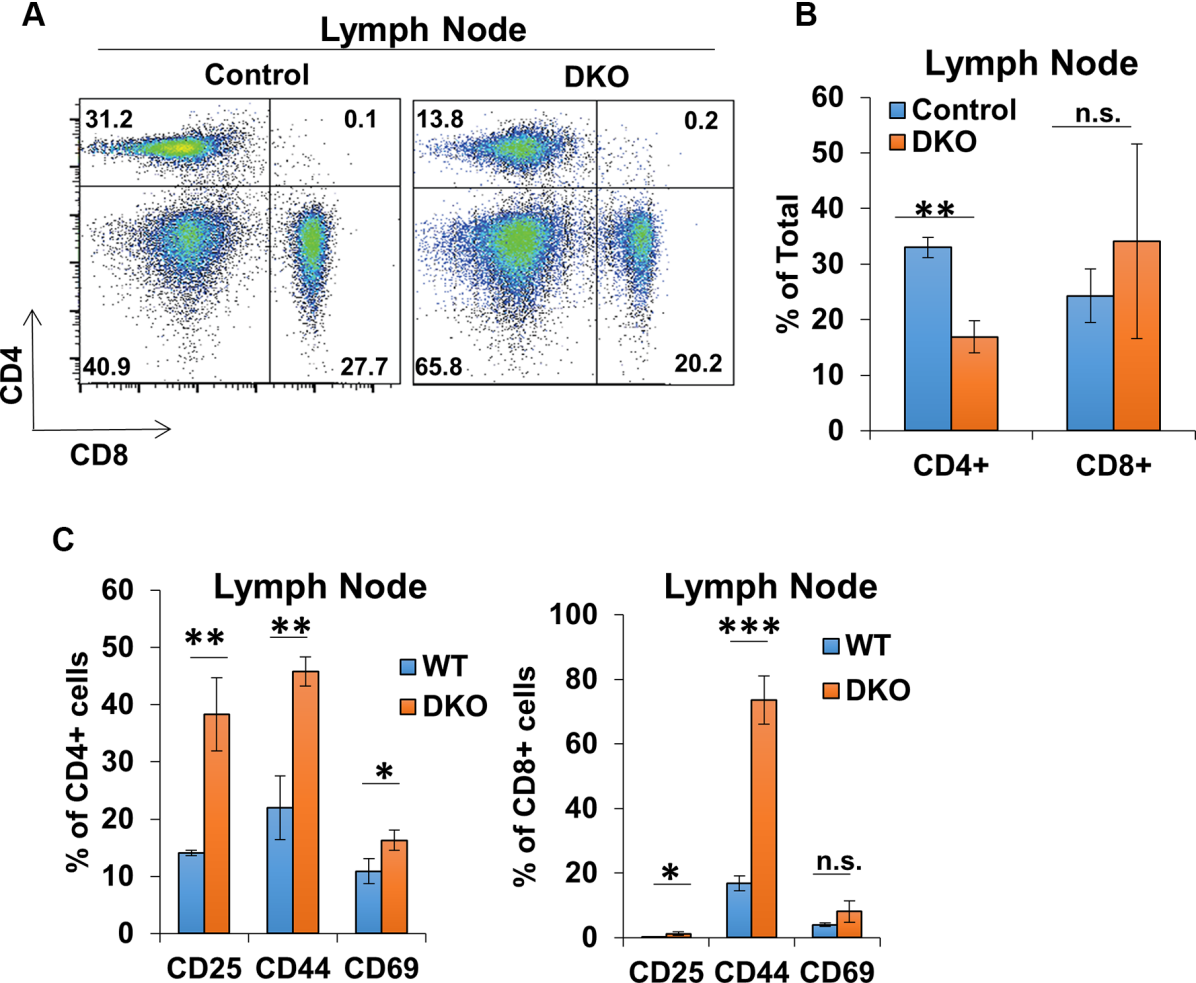
CD4-Cre induced CBL/CBL-B deletion leads to altered T-cell phenotype in lymph nodes (**A–B**) Representative FACS dot plots (A) and mean values of percentage of total lymph node cells stained with anti-CD4 and anti-CD8 (B) in Control and DKO mice; *n* = 3. (**C**) Quantification of the percentage of cells positive for markers CD25, CD44, and CD69 in lymph node CD4+ (left) and CD8+ (right) gated populations; *n* = 3. Data shown are mean +/− SD. ns, *p* ≥ 0.05; *p ≤ 0.05; **p ≤ 0.01; ***p ≤ 0.001; ****p ≤ 0.0001.

CBL/CBL-B deficiency in T-cells was previously shown to result in a constitutively-activated T-cell phenotype [15, 27, 30], as opposed to hyperactivity only upon extrinsic stimulation of T-cells in CBL-B-null mice [9, 15, 16]. To assess the impact of concurrent deletion of CBL/CBL-B in T-cells in CD4-Cre-bearing conditional DKO mice, we evaluated the activation status of peripheral T-cells in secondary lymphoid tissues. Compared to control, CD4+ and CD8+ T-cell populations contributed to a smaller percentage of the total splenic cells in DKO mice despite having overall greater numbers (Figure 4A and 4B). However, both CD4+ and CD8+ splenic T-cell populations in DKO mice exhibited lower levels of CD45RB and CD62L, as well as an increase in the ratio of cells positive for activation markers CD25, CD44 and CD69 as compared to control mice (Figure 4C and 4D). Examination of lymph node T-cell populations revealed a decrease in the percentage of CD4+ T-cells in DKO mice compared to control mice, similar to that seen in the spleen; however, the percentage of the CD8+ T-cell population appeared to be unchanged between control and DKO mice (Figure 5A and 5B). Also, similar to spleen, CD4+ and CD8+ T-cell populations in DKO lymph nodes showed a higher percentage of cells positive for activation markers compared to control mice (Figure 5C). Collectively, these findings lead to a clear conclusion that CD4-Cre induced concurrent CBL/CBL-B DKO leads to alleviation of negative regulatory mechanisms of T-cell activation resulting in constitutively-activated T-cells.

### CD4-Cre directed CBL/CBL-B deletion in Non-T-cell lineages

**Figure 6:**
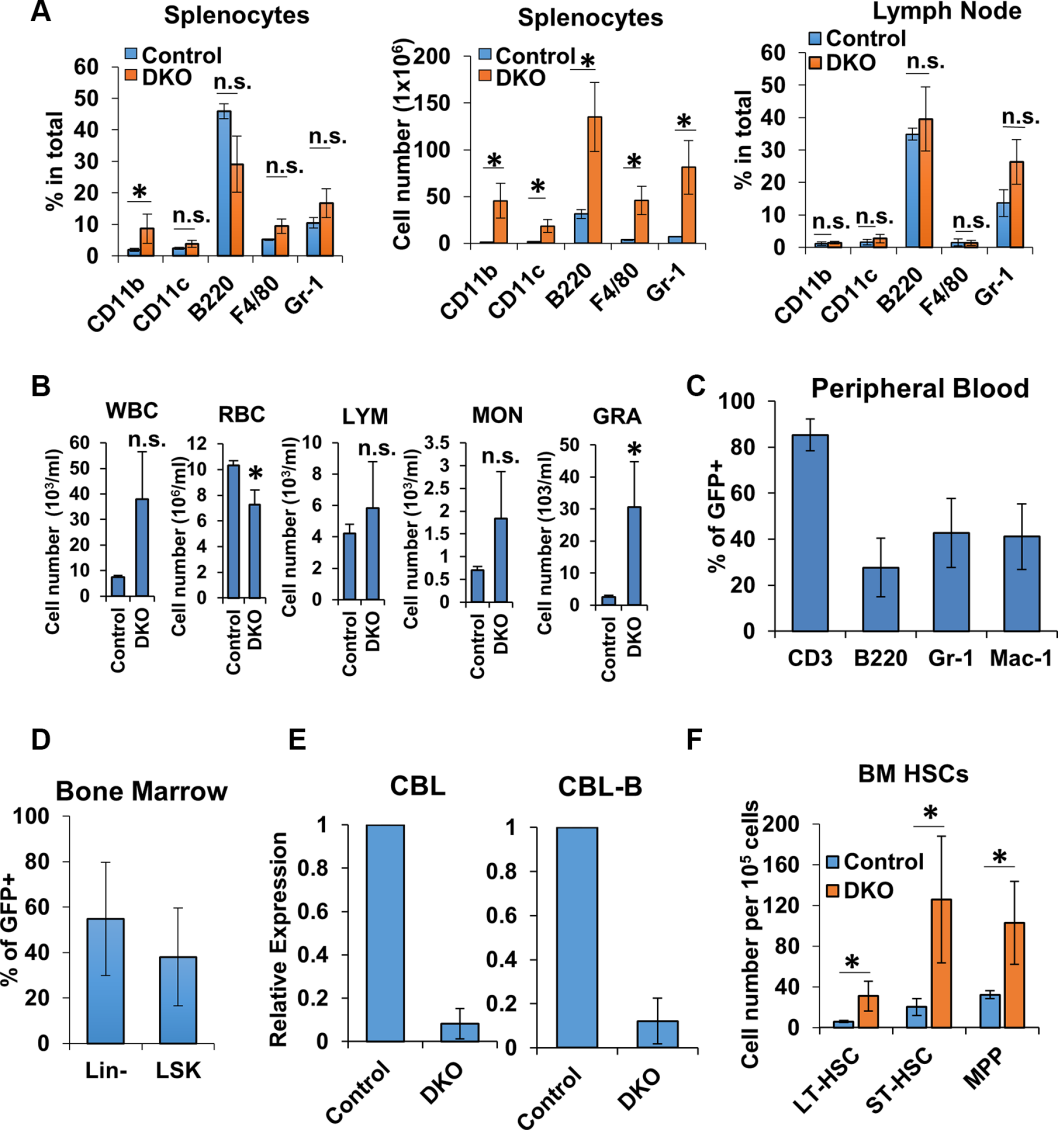
Non-T-cells lineages were impacted by CD4-Cre (**A**) FACS analysis of spleen (left and center) and lymph node (right) from Control and DKO mice for non-T-cell markers; *n* = 3. (**B**–**C**) Peripheral blood cell counts (B) and FACS analysis for the expression of GFP (C) of Control and DKO mice; *n* = 3. WBC, white blood cell; LYM, lymphocyte; MON, monocyte; GRA, granulocyte; RBC, red blood cell. (**D**) FACS analysis of bone marrow Lin- and LSK cells from DKO mice for GFP expression. (**E**) mRNA expression levels of CBL (left) and CBL-B (right) were analyzed in FACS-sorted Lin- cells of Control or DKO mice by quantitative real-time PCR. (**F**) FACS analysis of bone marrow cells from Control and DKO mice HSCs (LT-HSC, ST-HSC and MPP). Data shown are mean +/− SD of at least 3 mice replicates. ns, *p* ≥ 0.05; *p ≤ 0.05; **p ≤ 0.01; ***p ≤ 0.001; ****p ≤ 0.0001.

A dramatically-activated phenotype of T-cells in peripheral lymphoid tissues coupled with the result that CD4+ T-cells represented a smaller percentage of total cells in these tissues suggested the possibility that CD4-Cre induced DKO was associated with direct or indirect alterations in non-T-cell lineages. To investigate this possibility, we assessed the changes in the percentage and numbers of non-T hematopoietic lineage cells by FACS Supplementary Figure S1. Spleen and lymph nodes were collected from control and DKO mice, and B cells and myeloid cells were evaluated. Compared to control mice, DKO spleens showed an expansion of B cells and myeloid cells, evidenced by an increase in the absolute numbers of cells carrying CD11b, CD11c, B220, F4/80 or Gr-1 (Figure 6A). Similarly, the Gr-1^+^ population was also substantially, albeit not statistically significant, expanded in DKO lymph nodes (Figure 6A). Peripheral blood differential cell counts further demonstrated a dramatic increase in myeloid cell populations in DKO mice compared to Control (Figure 6B).

To determine whether the alterations of non-T hematopoietic lineage cells was due to cell intrinsic loss of CBL/CBL-B or indirectly due to an effect of extrinsic signals from activated T-cells, we carried out FACS-based analysis of GFP reporter gene expression to assess if CD4-Cre induced gene deletion was confined to T-cells as expected, or if non-T-cell lineages also showed evidence of gene deletion. As shown in Figure 6C, GFP was expressed in a majority of T-cells as anticipated; however, a subset of both B cells and myeloid populations were GFP+, indicative of unexpected deletion of CBL and CBL-B in non-T lineages.

Our results that CD4-Cre-mediated reporter gene deletion occurred in multiple non-T-cell lineages and myeloid cell proportion was increased in the peripheral blood of mice rendered DKO using CD4-Cre suggests the possibility that CD4-Cre may concurrently direct CBL/CBL-B deletion either in these lineages or in HSCs since CBL/CBL-B deletion in HSCs using MMTV-Cre leads to a myeloid-skewed expansion [36, 37]. We therefore assessed whether CD4-Cre directed GFP reporter gene expression is observed in HSCs from which all hematopoietic lineage cells are derived [58]. To address this, we assessed the GFP expression of lineage marker-negative (Lin cells; hematopoietic stem and progenitor cells) and Lin^−^ sca-1^+^ and c-Kit^+^ (LSK cells; HSC-enriched population) cells isolated from the bone marrow of DKO mice using FACS. Notably, both Lin^−^ and LSK populations in DKO mice contained a substantial subset of GFP+ cells (Figure 6D) and real-time PCR analysis demonstrated an over 90% reduction in CBL and CBL-B expression in FACS sorted GFP+ Lin- cells (Figure 6E). In addition, CD4-Cre induced CBL/CBL-B deletion leads to a significant expansion of long-term HSC (LT-HSC), short-term HSC (ST-HSC) and multipotent progenitors (MPP) cell populations compared to control mice (Figure 6F). This recapitulates the phenotype of mice with MMTV-Cre induced deletion of CBL and CBL-B in HSCs [36, 37]. These data lead us to conclude that CD4-Cre can direct gene deletion in a proportion of non-T hematopoietic lineages starting with the HSC stage.

### Expression of CD4 in HSC

**Figure 7:**
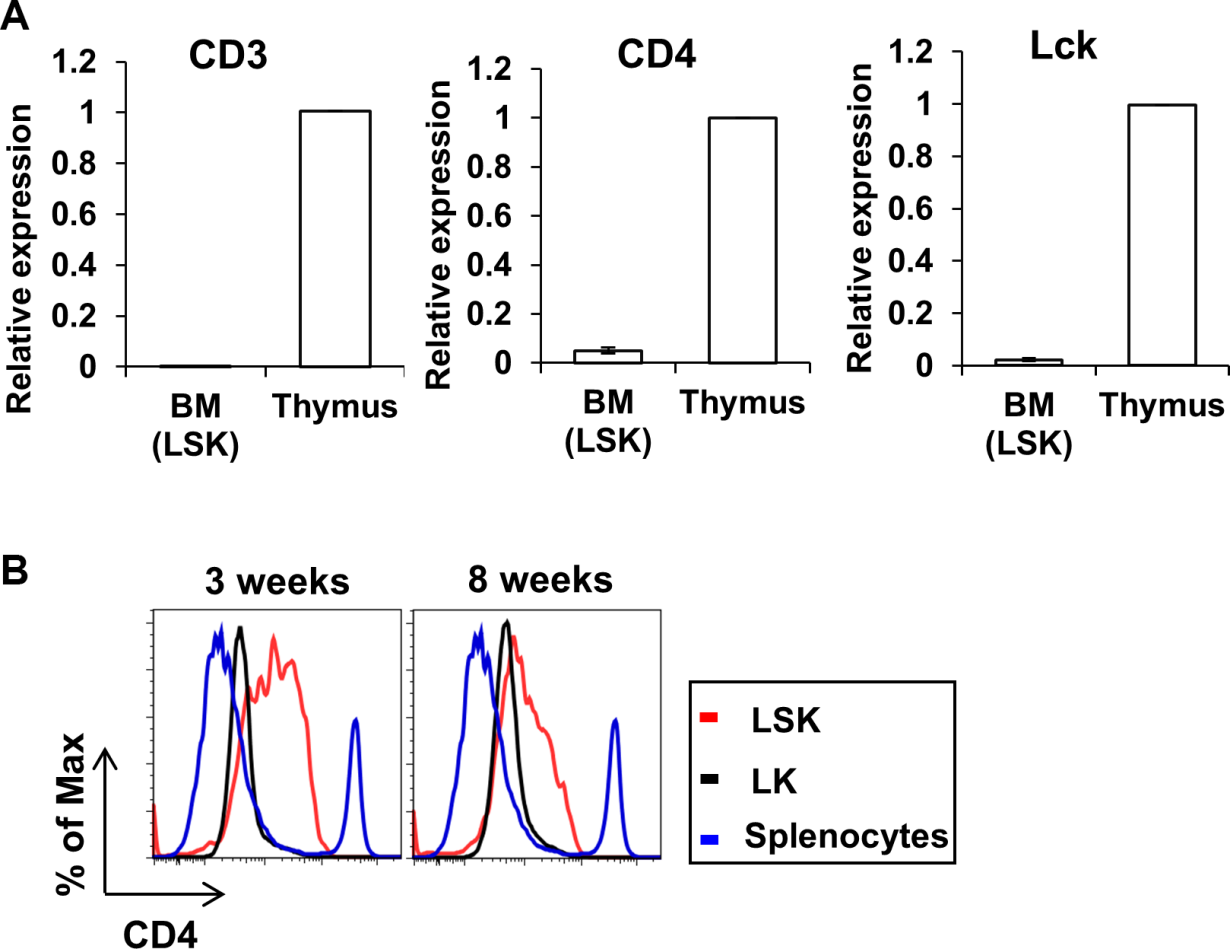
Expression of CD4 in HSC (**A**) BM cells were collected from 3 weeks old WT mice and Lin- cells were FACS sorted followed by mRNA purification. Expression levels of CD3 (left), CD4 (center) and Lck (right) were analyzed by quantitative real-time PCR. Expression of target gene in Lin- cells is normalized to thymus control. Data show mean+/− SD of 3 replicates. (**B**) BM cells were collected from 3 weeks (left) or 8 weeks (right) mice and labeled with stem cell markers and CD4. Splenocytes were used as positive control for the expression of CD4. Data shown is one representative data of three repeats.

CD4 is considered a T-cell marker and CD4 promoter elements have been used extensively in genetic studies to direct T-cell specific gene expression or deletion [59–68]. Given our results that CD4-Cre can direct gene deletion in HSCs, and old reports that a small proportion of HSCs express low levels of CD4 detectable with antibodies [69, 70], we next addressed if CD4 was indeed expressed in HSCs using a more sensitive quantitative real-time PCR (qPCR) assay (Figure 7A). HSCs (LSK cells) were sorted from WT BM cells and mRNA was analyzed for CD4 expression by qPCR, with T-cell specific CD3 [71] as a positive control for any T-cell contamination. Compared to the undetectable expression of CD3, a low but detectable level of CD4 mRNA expression was observed in HSCs, together with a detectable expression of Lck, a key signal transducer downstream of CD4 [72]. Furthermore, using WT BM cells from 3-week and 8 week old mice, we performed FACS analysis to evaluate the expression of CD4 on HSCs and hematopoietic progenitors (Figure 7B). Notably, compared to splenocytes (used as a positive control), LSK cells express an easily-detectable level of CD4 on the cell surface, consistent with previous reports [69, 70]. Collectively, these data suggest that CD4-Cre, by virtue of authentic expression of CD4 in a subset of HSCs and hematopoietic progenitors, can direct gene deletion in early hematopoietic lineages including HSCs, which can thereby add complexities to phenotypes assigned to T-cell specific gene deletion directed by CD4-Cre.

## DISCUSSION

CBL-family ubiquitin ligases are essential negative regulators of T-cell activation that impinge on anergy induction program. Tight regulation of T-cell activation and immunological tolerance are essential for effective defense against foreign antigens and immune surveillance against cancer without mounting autoimmunity to self-antigens or producing protracted inflammatory diseases following infections. Previous models have failed to accurately elucidate the role that CBL proteins play in a T-cell-specific manner as these models utilized a CBL-B null background [13, 14], which leads to the altered and/or enhanced function of B cells [19], macrophages [20, 21], mast cells [22], neutrophils [23, 24], and NKT-cells [25]. Particularly in the context of tumorigenesis, the available CBL-B-null model has not been suitable for *in vivo* studies to assess the tumor cell-intrinsic roles of CBL proteins since CBL-B-null mice reject tumors due to their activated CD8+ T-cells [5, 23] and NK cells [24]. Studies described here describe the establishment and characterization of the first inducible model of CBL-B deletion. By crossing this new model to a previously established and sparingly-used CBL-^flox/flox^ mouse, we have now established the first fully-conditional model of tissue-specific CBL/CBL-B DKO mouse model that will help overcome the current barrier in understanding the redundant roles of CBL proteins in physiological systems as well as in oncogenesis.

We established the functionality of the new floxed CBL-B alleles engineered in the mouse genome by demonstrating the generation of a whole-body CBL-B null mouse by crossing the CBL-B floxed mice with EIIA-Cre transgene, which is known to drive Cre expression very early during development, including in germ cells [73]. Analyses of T-cells of these mice recapitulated the hyper-responsiveness to TCR engagement comparable to that seen using T-cells of previously generated whole-body KO mice [15] (Figures 4 and 5). As a proof of principle of tissue-specific concurrent deletion of CBL and CBL-B in the CBL-^flox/flox^/CBL-B-^flox/flox^ mice we generated, we chose to induce a DKO in T-cells since prior studies using Lck-Cre-driven deletion of CBL-^flox/flox^ gene on a CBL-null background demonstrated that CBL and CBL-B function redundantly in T-cells and the DKO mice exhibit a profound and lethal inflammatory disease [30]. We used a CD4-Cre driver for these studies as this Cre has been extensively used for T-cell-specific deletion of conditionally-targeted genes [59–68]. CD4-Cre mediated DKO led to severe systemic, autoimmune/inflammatory disease with mice becoming moribund as early as 10 weeks of age accompanied by immune cell infiltration in multiple organs including liver, brain, kidney, and lung (Figure 2C, 2E). Notably, our findings differ from those of the previous T-cell DKO studies [30] in that we also observed immune cell infiltration in brain, kidney, and lung.

CD4-Cre expression is expected to begin at DP stage during thymic T-cell development [74]. While this is considerably later than that of Lck-Cre used in the previous studies, which is active as early as DN3 stage of double-negative thymocytes [31], T-cell development was altered in the CD4-Cre driven DKO mice with a reduction in total thymocyte numbers and skewing of thymocyte populations (Figure 3A–3D). These features and the increase in the DN4 thymocyte populations are similar to those seen in mice with Lck-Cre induced CBL deletion on a CBL-B-null background [31]. An impact on the DN4 populations is somewhat unexpected but consistent with the known role of CBL in negatively regulating the pre-TCR signaling [75]. This feature may be reflection of CD4-Cre mediated CBL/CBL-B deletion at earlier stages of hematopoiesis as discussed below. As demonstrated in previous work [31], decrease in the DP thymocyte population can be attributed to alleviation of the negative regulation of TCR signaling, resulting in accentuation of TCR signal strength, which converts a positive into a negative T-cell selection.

We also show that CD4-Cre mediated deletion of CBL and CBL-B genes leads to constitutive activation of peripheral T-cell populations in the spleen and lymph node as demonstrated by the changes in the expression levels of activation-related markers (Figure 4C, 4D and Figure 5C). These data show that concurrent CBL and CBL-B deletion during T-cell development using CD4-Cre largely recapitulates the T-cell activation and systemic immune cell infiltration phenotype previously described, providing direct support that the new CBL-B-flox model and its combination with the existing CBL-flox model will allow controlled, tissue-specific deletion of CBL and/or CBL-B in specific cell types. Importantly, the newly-validated floxed models will allow, for the first time, a dissection of the specific as well as redundant roles of CBL and CBL-B to fully explore their roles in adult mammalian tissue function and pathology without the inherent developmental issues associated with the whole body CBL KO mice [76–78], immune hyperactivity of CBL-B-null mice [19], and importantly eliminate the issue of embryonic lethality of germline double KO mice. Importantly, this model will allow concurrent CBL and CBL-B deletion in non-hematopoietic tissues to understand the role of these proteins in physiology and tumorigenesis, without spontaneous tumor rejection.

While deletion of CBL and CBL-B in T-cells (Figure 2A, 2B) was expected, the alteration of DN thymocyte populations, a more aggressive phenotype of CD4-Cre mediated DKO mice compared to that of previously described Lck-Cre driven DKO [30], and a marked decrease in the relative proportions of peripheral CD4+ T-cells suggested that CD4-Cre driven CBL/CBL-B DKO may also occur in other hematopoietic lineages, a possibility not considered in previous studies. Indeed, we demonstrated that CBL and CBL-B deletion was present in other hematopoietic lineages (Figure 6A). Given our previous studies in which MMTV-driven deletion of CBL in a small percentage of HSCs led to a myeloid-skewed expansion of peripheral blood cell lineages and HSC expansion [36, 37], one possible explanation for these discrepancies was that CBL and CBL-B are deleted in a certain proportion of HSCs, which consequently manifests as CBL/CBL-B deletion in other non-intended lineages within the hematopoietic system. This notion is consistent with previous reports, which have received little attention in the context of the use of CD4-Cre for gene deletion in which CD4 expression was shown on a small subset of HSCs using antibody-based staining [69, 70]. We provide further support for this idea using real-time PCR and FACS analysis demonstrating the expression of the Cre reporter (GFP) in HSCs as well as differentiated hematopoietic cell populations (Figure 6C, 6D, 6E). CD3, a marker of T-lineage cells was not detectable in HSCs, reducing the likelihood of the CD4 signals arising from any lingering T-cell contamination in our LSK population. Moreover, the Lin- Sca-1- c-Kit+ (LK) population, which represents the more committed myeloid progenitor population [58], exhibited a lower level of CD4 expression level (Figure 7B) compared to LSK cells, suggesting that CD4 expression in immature hematopoietic stem/progenitors is a transient event.

The role of CD4 expression in HSCs is unclear. The likely explanation for why a potentially-transient CD4-Cre directed deletion of CBL and CBL-B in a small percentage of HSCs would manifest more strongly in our studies is that HSCs with loss of CBL and CBL-B acquire a robust proliferative advantage, as has been demonstrated in previous studies [36, 37, 79]. The apparent lack of any robust HSC-based phenotype in previous studies [59–68] could reflect a lack of consideration of such a phenotype due to lack of a proliferative advantage of the targeted gene deletion or a negative impact on proliferation. Regardless of the role of CD4 in HSCs, our results suggest caution in designing CD4-Cre based deletion strategies and assigning the phenotypes solely to gene deletion in T-cells.

Overall, our studies establish a new model of inducible CBL/CBL-B deletion that should allow the immune vs. non-immune cell-intrinsic roles of these key but functionally redundant negative regulators of tyrosine kinase signaling to be determined. Further, for the first time, the new model will allow the role of CBL and CBL-B to be determined in tumorigenesis without the current lack of feasibility of such studies due to tumor rejection from germline deletion of CBL-B using existing models.

## MATERIALS AND METHODS

### Mice

In order to generate CBL-B conditional knockout mice, a CBL-B conditional knockout construct was engineered using “recombineering” technique (Figure 1A) [80]. The Clone Finder software (NCBI database) was used to search the NIH's C57BL/6J (B6) mouse BAC library (at the Children's Hospital Oakland Research Institute). We identified clones [RP23-456D16, RP23-122H13, RP24-361F9, RP24-98B21] that contain the mouse CBL-B gene. A series of “recombineering” reactions [80] were used to retrieve a 10.5 kb fragment of the BAC DNA containing the first and second exons of CBL-B into a plasmid (with negative selection marker), allowing us to introduce two loxP (Cre recombinase recognition) sites flanking this region. Immediately preceding the second loxP site an engineered FRT-Neo-FRT selection cassette was inserted which confers G418 resistance to transfected ES cells. FRT sites allow removal of the Neo gene using FLP recombinase thus keeping alterations of the gene locus to a minimum. The correct arrangement and sequence of targeted gene segments at each step have been verified by PCR analysis and sequencing. A NotI linearized targeting vector was submitted to the Mouse Genome Engineering Core Facility at UNMC for electroporation into a C57BL/6-derived ES cell line. Southern blot hybridization with probes located outside the 5′ and 3′ boundaries of the targeted region was used for screening of 5′ and 3′ boundaries of the targeted region to identify targeted ES clones after G418 and guancyclovir selection.

C57BL/6 ES clones in which the CBL-B gene was correctly targeted were used to produce chimeric mice using blastocyst injection. Chimeras were mated to B6 mice to test the germline transmission of the targeted CBL-B allele and verified using Southern blot hybridization and PCR analysis of tail-derived genomic DNA.

Next, heterozygous CBL-B targeted mice (CBL-B^f-Neo/+^) were intercrossed to generate homozygous CBL-B targeted mice (CBL-B^f-Neo/f-Neo^). The homozygous CBL-B targeted mice were mated to B6; SJLTg(ACTFLPe)9205Dym/J mice which express the enhanced FLP1 recombinase (FLPe) from the ubiquitously expressed human ACTB (beta actin) promoter to remove the FRT-flanked Neo gene. Heterozygous CBL-B targeted, FLPe transgene-positive mice were crossed to C57BL/6J (wild-type mice) in order to generate heterozygous CBL-B-floxed, FLPe transgene-negative (*CBL-B*^*fl*/+^) mice. These heterozygous mice were mated to produce homozygous CBL-B floxed mice (CBL-B^f/f^). To examine the functionality of the loxP site in the engineered mice and generate CBL-B null mice, we crossed the CBL-B floxed mice to B6.FVB-TgN (EIIa-Cre) C5379Lmgd, which expresses Cre ubiquitously from the EIIa promoter. Heterozygous *CBL-B*-targeted, EIIa-*Cre* transgene-positive mice were crossed to C57BL/6J (wild-type) mice to generate heterozygous *CBL-B-*deleted, *Cre* transgene-negative (*CBL-B*^+/−^) mice, which were used to produce *CBL-B*^−/−^ mice.

CBL^flox/flox^ [30], CBL-B^flox/flox^, CD4-Cre [Tg(Cd4-cre)1Cwi/BfluJ] (The Jackson Laboratory), and Cre mT/mGFP reporter [Gt(ROSA)26Sortm4(ACTB-tdTomato,-EGFP)Luo/J] (The Jackson Laboratory) strains were maintained on a C57/Bl6 background under specific pathogen-free conditions (Supplementary Table 1) and genotyped using tail DNA PCR with the primers specified in (Supplementary Table 2). All mouse experiments were approved by the UNMC IACUC.

### Tissue preparation and FACS analysis

Single cell suspensions were made from spleen, thymus, and lymph node by mashing tissue through a 40 μm cell strainer and RBCs were lysed using ACK Lysing Buffer for 10 min at RT. For cell analysis and sorting, cells were immuno-stained for 20 min at 4°C in FACS buffer (PBS-1% BSA). The following antibodies were procured from eBioscience: CD4 (RM4-5); CD62L (MEL-14); CD45RB (C363.16A); CD69 (H1.2F3); B220 (RA3-6B2); F4/80 (BM8). The following antibodies were procured from BD Biosciences: CD8 (53-6.7); CD117 (2B8); CD11b (M1/70); anti-CD11c (HL3); Gr-1 (RB6-8C5). CD25 (PC61) and CD44 (IM7) were from Biolegend.

Whole bone marrow cell suspensions were prepared from femurs and tibiae. For stem and progenitor cell analysis and sorting, mature hematopoietic cells (lineage-positive cells) were labeled with antibodies against CD5, B220, CD11b, Gr-1, and 7-4 (mouse lineage depletion kit; Miltenyi Biotechnology) and magnetically depleted using the autoMACS (Miltenyi Biotechnology). Lineage-negative cells were then stained with antibodies followed by cell analysis or sorting. Flow cytometry was performed on a BD LSRII or Aria II at the UNMC Flow Cytometry Research Facility. Data were analyzed using FlowJo software (Tree Star). Cell populations were defined as follows [81] LT-HSC: CD34 FLT3^−^ Lin^−^ Sca-1^+^ c-Kit^+^; ST-HSC: CD34^+^ FLT3^−^ Lin- Sca-1^+^ c-Kit^+^; MPP: CD34^+^ FLT3^+^ Lin- Sca-1^+^ c-Kit^+^; LSK: Lin^−^ Sca-1^+^ c-Kit^+^; CMP: CD16^−^ CD34^+^ Lin^−^ Sca-1^−^ c-Kit^+^; GMP: CD16^+^ CD34^+^ Lin- Sca-1^−^ c-Kit^+^; MEP: CD16^−^ CD34^−^ Lin^−^ Sca-1^−^ c-Kit^+^; CLP: IL-7R^+^ FLT3^+^ Lin- Sca-1^low^ c-Kit^low^.

### Western blotting

For CBL-B protein expression analysis, splenocytes were lysed using lysis buffer (1 M Tris pH 7.5, 5 M NaCl, 10% Triton, 100 mM VO4, 1 M NaF, 50 mM PMSF). The following antibodies for western blotting were procured from commercial sources: Rabbit monoclonal antibody (mAb) anti-CBL-B (Clone D3C12, Cell Signaling Technology).

### ELISA

Splenocytes were cultured in RPMI supplemented with 10% FBS in triplicates in 96 U-bottom wells (2 × 10^6^ cells/well) precoated with 10 μg/ml plate-bound anti-CD3ε (145-2C11) in the presence or absence of 1 μg/ml soluble anti-CD28 (37.51) for 48 h at 37°C. Culture supernatants were collected and the IL-2 concentration measured by Quantikine ELISA kit (R&D systems), according to the manufacturer's instructions.

### RNA isolation and quantitative real-time PCR analysis

RNA extracted from FACS-sorted cells (RNAqueous -Micro kit, Life Technologies) was reverse-transcribed (QuantiTect kit, Qiagen) and subjected to quantitative real-time PCR (QuantiTect SYBR Green Kit, Qiagen) on a BioRad CFX96 thermocycler, following the manufacturer's instructions. Primers are listed in Supplementary Table 3.

### Histopathology

Organs were formalin-fixed, dehydrated in 70% EtOH, paraffin-embedded and H&E stained. Whole blood CBCs were performed on a Scil Vet abc Animal Blood Counter (Scil Animal Care).

### Statistics

Quantified results of qPCR and flow cytometry were compared between groups using Student's *t* test, and are presented as mean +/− SD, with *p* ≤ 0.05 deemed significant. Statistical analysis and graphical representation of data were performed using GraphPad Prism version 4.0c (GraphPad Software, San Diego, CA). Data shown are mean +/− SD. ns, *p* ≥ 0.05; *p ≤ 0.05; **p ≤ 0.01; ***p ≤ 0.001; ****p ≤ 0.0001.

## SUPPLEMENTARY MATERIALS FIGURE AND TABLES


