# Supplementary material for: A novel CBL-Bflox/flox mouse model allows tissue-selective fully conditional CBL/CBL-B double-knockout: CD4-Cre mediated CBL/CBL-B deletion occurs in both T-cells and hematopoietic stem cells
Source: Oncotarget. 2016 Jun 3;7(32):51107–23. doi: 10.18632/oncotarget.9812 (PMC5239462; doi:10.18632/oncotarget.9812)
Supplement: Supplementary file 1 [file oncotarget-07-51107-s001.pdf]

A novel *CBL-B$^{flox/flox}$* mouse model allows tissue-selective fully conditional *CBL/CBL-B* double-knockout: CD4-Cre mediated *CBL/CBL-B* deletion occurs in both T-cells and hematopoietic stem cells

Supplementary Materials

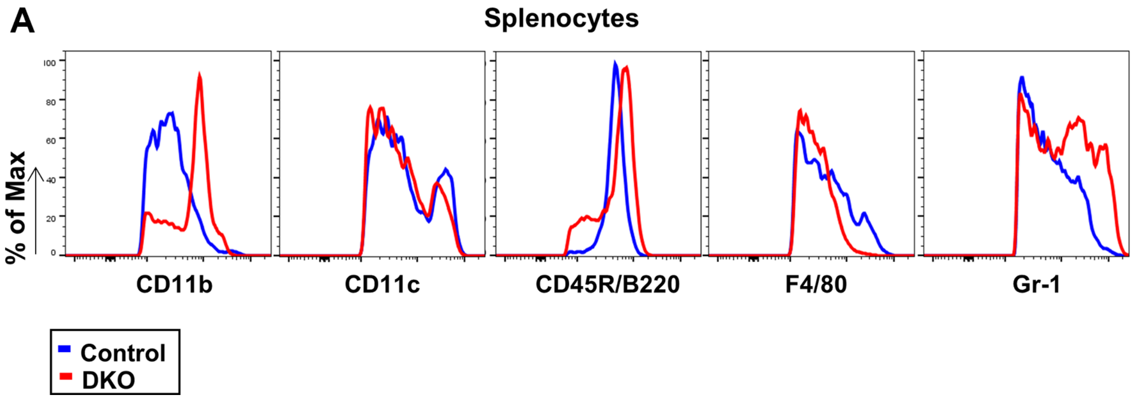

Supplementary Figure S1: Splenic Non-T cell lineages were impacted by CD4-Cre. (**A**) Representative histograms for FACS analysis of non-T cell marker expression in the spleen of Control and DKO mice.

Supplementary Table S1: Genotypes of mice used in this paper

Strain designation	Genotype
WT	C57/B6
Control	CBL$^{flox/flox}$ CBL-B$^{flox/flox}$
CBL/CBL-B DKO	CBL$^{flox/flox}$ CBL-B$^{flox/flox}$ CD4-Cre$^{Tg/0}$
CBL/CBL-B DKO mT/mGFP	CBL$^{flox/flox}$ CBL-B$^{flox/flox}$ CD4-Cre$^{Tg/0}$ mT/mG

Supplementary Table S2: Genotyping primers

Target Allele	Forward 5′-3′	Reverse 5′-3′
CBL floxed	GTGGTGGCTTGCAATTATAATCCTACCACT-TAGG	GTTTGAGATGTCTGGCTGTGTACAC-GCG
CBL-B floxed	GGCAGAACCACTGAGACACATTTA	GGCTGCCAAACTGCTACCCAGGAG
CD4-Cre	GCGGTCTGGCAGTAAAAACTATC	GTGAAACAGCATTGCTGTCACTT
Rosa 26 – mT/mGFP	CTCTGCTGCCTCCTGGCTTCT	TCAATGGGCGGGGGTCGTT

Supplementary Table S3: Primers used for quantitative real-time PCR

Target	Forward 5′-3′	Reverse 5′-3′
CBL	AGCTGATGCTGCCGAATTT	TTGCAGGTCAGATCAATAGTGG
CBL-B	GGAGCTTTTTGCACGGACTA	TGCATCCTGAATAGCATCAA
CD4	GAGAGTCAGCGGAGTTCTC	CTCACAGGTCAAAGTATTGTTG
LCK	CGCATGGTGAGACCTGACAA	TCCGAAGGTAGTCAAACGTGG
CD3	TGCCTCAGAAGCATGATAAGC	GCCCAGAGTGATACAGATGTCAA
GAPDH	CCTGGAGAAACCTGCCAAGTATG	AGAGTGGGAGTTGCTGTTGAAGT
